# The impact of bipolar spectrum disorders on professional functioning: A systematic review

**DOI:** 10.3389/fpsyt.2022.951008

**Published:** 2022-08-24

**Authors:** Monika Dominiak, Piotr Jażdżyk, Anna Z. Antosik-Wójcińska, Magdalena Konopko, Przemysław Bieńkowski, Łukasz Świȩcicki, Halina Sienkiewicz-Jarosz

**Affiliations:** ^1^Department of Pharmacology, Institute of Psychiatry and Neurology, Warsaw, Poland; ^2^Department of Affective Disorders, Institute of Psychiatry and Neurology, Warsaw, Poland; ^3^Chair and Department of Experimental and Clinical Physiology, Laboratory of Centre for Preclinical Research, Medical University of Warsaw, Warsaw, Poland; ^4^Department of Psychiatry, Medical University of Warsaw, Warsaw, Poland; ^5^First Department of Neurology, Institute of Psychiatry and Neurology, Warsaw, Poland

**Keywords:** bipolar spectrum, manic or depressive episode, bipolar disorder, personality disorders, employment outcomes, professional functioning

## Abstract

**Aims:**

The impact of bipolar spectrum (BS) disorders on professional functioning has not been systematically reviewed yet. Since even subsyndromal symptoms may disturb functioning, the determination of the prognostic value of the spectrum of bipolarity for employment seems extremely relevant. The aim of this study was to assess the impact of BS disorders on professional functioning.

**Materials and methods:**

A systematic review of the literature (namely, cohort and cross-sectional studies) investigating a link between BS disorders and employment was performed in accordance with PRISMA guidelines. BS was defined based on the concept of two-dimensional BS by Angst. Occupational outcomes and factors affecting employment were evaluated as well.

**Results:**

Seventy-four studies were included. All disorders comprising BS had a negative impact on occupational status, work performance, work costs, and salary, with the greatest unfavorable effect reported by bipolar disorder (BD), followed by borderline personality disorder (BPD), major depressive disorder (MDD), and dysthymia. Employment rates ranged from 40 to 75% (BD), 33 to 67% (BPD), 61 to 88% (MDD), and 86% (dysthymia). The factors affecting employment most included: cognitive impairments, number/severity of symptoms, namely, subsyndromal symptoms (mainly depressive), older age, education, and comorbidity (substance abuse, personality disorders, anxiety, depression, ADHD, PTSD).

**Conclusion:**

Bipolar spectrum symptoms exert a negative impact on professional functioning. Further evaluation of affecting factors is crucial for preventing occupational disability.

## Introduction

Economic inactivation has been proved to be an emerging problem within the last decade (1). This problem, visible especially among young people aged 20–34, has grown over recent years, becoming a major socioeconomic and medical challenge (2). According to a conservative estimate, the costs generated by the disengagement of young people from the labor market amount to €153 billion, which corresponds to 1.2% of European GDP (3). It has been estimated that approximately 18.3% of young people aged 20–34 are neither employed nor involved in education or training (4). Possible reasons for such a situation include psychological and sociodemographic factors. In particular, the factors contributing to high risks of unemployment encompass migration background, low education level, remote areas of living, parents with a history of unemployment, as well as female gender (3). It should be noted, however, that it is often difficult to differentiate between sociodemographic factors that lead to economic inactivation and those which are simply correlated with such status (5, 6). It seems that one of the main variables affecting occupational activities may be the prevalence of mood disorders, also those that do not fit to international classifications and criteria for diagnosing depression or bipolar disorder (BD). There is a great proportion of patients with a clinical picture resembling depressive disorder but showing at the same time discrete features of bipolar symptoms (7). The onset of these disorders is most typically in early adulthood.

**FIGURE 1:**
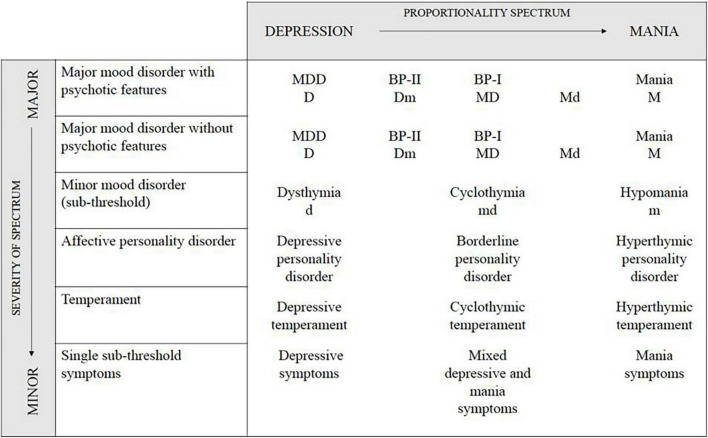
The concept of two-dimensional affective spectrum based on Angst (10). The horizontal axis represents the first dimension of this model–a continuum of proportional mood spectrum beginning with depression, through three bipolar subgroups, and ending with mania. The second dimension (vertical axis) refers to the severity of symptoms, which range from a major mood disorder, to affective personality disorder, temperament, and finally to single symptoms of bipolarity with severity close to normal. MDD, major depressive disorder; BP-I (MD), bipolar disorder type I; BP-II (Dm), bipolar disorder type II; D, major depression; d, mild depression; M, mania; m, hypomania; Md, mania with mild depression; md, minor bipolar disorder.

The concept of the bipolar spectrum (BS) has been widely used in psychiatric terminology for almost three decades. Historically, it is a step back to Kraepelin’s manic-depressive insanity, in which mania and depression would be two parts of the same episode. With the release of the Diagnostic and Statistical Manual of Mental Disorders (DSM-III) (8) in the 1980s, Kreapelin’s manic-depressive insanity was divided into a broad concept of major depressive disorder (MDD) and a rump concept of BD. This description remained unchanged until the definitions of BS were created by Goodwin and Jamison (9), Angst (10), and Akiskal and Pinto (11). According to the above-mention researchers, BS would include not only classical but also milder forms of BD that do not fulfill diagnostic criteria described in the International Classification of Diseases-10 (ICD-10) (12) or DSM-V (13). Angst (10) published his concept of two-dimensional BS, which is believed to reduce the under-diagnosis of bipolarity. In its first dimension, this model presents a continuum of proportional mood spectrum beginning with depression, through three bipolar subgroups and ending with mania. The second dimension refers to the severity of symptoms, which range from a major mood disorder, to affective personality disorder, temperament, and finally to single symptoms of bipolarity with severity close to normal (Figure 1).

According to the systematic review, the lifetime prevalence of BD using non-uniform criteria ranges from 0.1 to 7.5%, while using stricter criteria and consistent methodology it ranges from 0.5 to 2.1% (14). Similarly, the estimated prevalence of BS varies, depending on the stringency of the diagnostic criteria and the concept of BS adopted, between 2.4 and 15.1% (broad spectrum disorder) and 2.4–4.4%, with the latter estimate unlikely to include MDD and dysthymia (14). Since BD presentations even at a subsyndromal level can cause distress, impair quality of life, and cause negative social consequences, the identification of patients with BS disorders is crucial, both at a clinical and socioeconomic level (15, 16). Employment outcome in affective disorders is important but still an under-investigated area (17). Although several studies have assessed the impact of BD or MDD on occupational status, the influence of BS has not been investigated in a systematic review till now.

The aim of the present systematic review was to analyze the data from observational studies regarding professional functioning in people with BS disorders. Specifically, we aimed at answering two main questions: (1) what is the impact of a given BS disorder on employment outcomes (i.e., employment rate, performance at work, salary, and labor costs) in comparison to both the general population and each other; (2) what factors are associated with employment outcomes in individuals with BS disorders.

## Materials and methods

The present systematic review was performed according to Preferred Reporting Items for Systematic Reviews and Meta-Analyses (PRISMA) guidelines (18). The ethical approval and the participants’ consent for their data usage in the research were not necessary either, as this is a review study.

### Search strategy

In September 2020 (with the updates in September 2021 and July 2022), two independent reviewers (MD and PJ) investigated the PubMed, PsycInfo, and Embase Databases (starting from the earliest to the most recent entries) for relevant articles. The search was performed among titles and abstracts by using a combination of the following sets of keywords: (set 1): “bipolar spectrum OR bipolar disorder OR bipolar OR bipolar illness OR depression OR mania OR hypomania OR dysthymia OR cyclothymia OR hyperthymia OR borderline personality disorder (BPD) OR emotionally unstable personality disorder OR depressive personality disorder OR hyperthymic personality disorder OR depressive temperament OR cyclothymic temperament OR cyclothymic trait OR cyclothymic disorder OR hyperthymic temperament; AND (set 2) employment OR unemployment OR occupation OR professional functioning.” Only studies written in English were considered. Search results were downloaded into EndNote version X9. After filtering the duplicates, titles and abstracts were screened independently by two reviewers to identify relevant papers. Any disagreements were resolved by consensus, or, if needed, by a third reviewer. Follow-up citations of retrieved studies were scanned for other relevant studies. Additionally, we also searched gray literature sources: ProQuest Dissertations and Theses Online, The Gray Literature Report from the New York Academy of Medicine, and OpenGrey.

### Eligibility criteria

For the purpose of this study, we used the concept of two-dimensional BS as defined by Angst (10). We also defined “employment” as paid work.

The inclusion criteria were as follows: (1) adult individuals with at least one condition: BD-I, BD-II, MDD, hypomania, mania, dysthymia, hyperthymia, cyclothymia, states with severe mania and minor depression, depressive/borderline/hyperthymic personality disorder, depressive/cyclothymic/hyperthymic temperament, subthreshold depressive, minor bipolar, or mania symptoms; (2) the presence of at least one of the conditions mentioned above and at least one parameter related to professional functioning; (3) observational (non-interventional) studies of any design; (4) publication in English.

The exclusion criteria were: (1) no distinction in the statistical analysis provided between patients with given disorders; (2) assessment of the influence of professional activity on the course of the illness or therapeutic value of employment; (3) focus only on the quality of life or functional status; (4) interventional studies or reviews.

### Data extraction

Two independent co-authors reviewed full articles and then compared their findings to reach a consensus. Any discrepancies were resolved by a third co-author (AA-W) until a final list of studies pertaining to the evaluation was compiled. The relevant studies evaluating the link between employment outcomes and BS disorders were collected. The following data were extracted: study sample (sample size, type of disorder, control group, if available), demographic and clinical characteristics (if available), study design, duration of follow-ups (if available), employment outcomes (any of the following: employment rate, presenteeism, absenteeism, earnings, labor costs), and clinical or demographical factors associated with professional functioning (if available).

### Risk of bias (quality) assessment

After investigating several rating systems for the evaluation of the quality of observational studies [such as the Newcastle−Ottawa System (NOS) protocol or the Risk of Bias Assessment Tool for Non-randomized Studies (RoBANS)], we found them inappropriate for this review. We developed a rating tool based on the tools quoted above, yet tailored for the aims of this review. The rating tool was tested by scoring 20 articles. Each article was scored independently by two reviewers. A third reviewer was consulted for opinion in case of disagreement. The score ranged from 1 to 5 stars. One star was given for the following criterion: (1) sample size > 100; (2) representativeness of the sample; (3) comparability of the control group (if the control group was present) or possible confounders reported in detail (demographic and clinical characteristics of participants); (4) the quality of measurement methods (i.e., measurements of work functioning, assessment tool for affective symptoms); (5) longitudinal design. Consequently, only longitudinal studies could receive a maximum of five stars. The quality of the studies did not constitute a reason for excluding them from completing the present review.

### Strategy for data synthesis

Search results from Endnote X9 were transferred to RevMan5. Heterogeneity was evaluated visually on the Forest plot and statistically by using the Chi-square, I^2^, and Tau^2^. However, due to the differences in the study design, outcome measures, as well as varying study populations and socioeconomic backgrounds, a majority of outcomes could not be pooled and meta-analyzed. The only variable of consistent measure–employment rate–was further evaluated by using a random effect model and subgroup analysis with regard to the severity of affective symptoms and study design. If at least two studies from a given country reported the employment rate, the pooled mean was estimated. In order to estimate publication bias, funnel plots of precision were evaluated.

Outcomes and factors other than employment rates that affected professional functioning were described together and summarized.

## Results

### Included study characteristics

**FIGURE 2:**
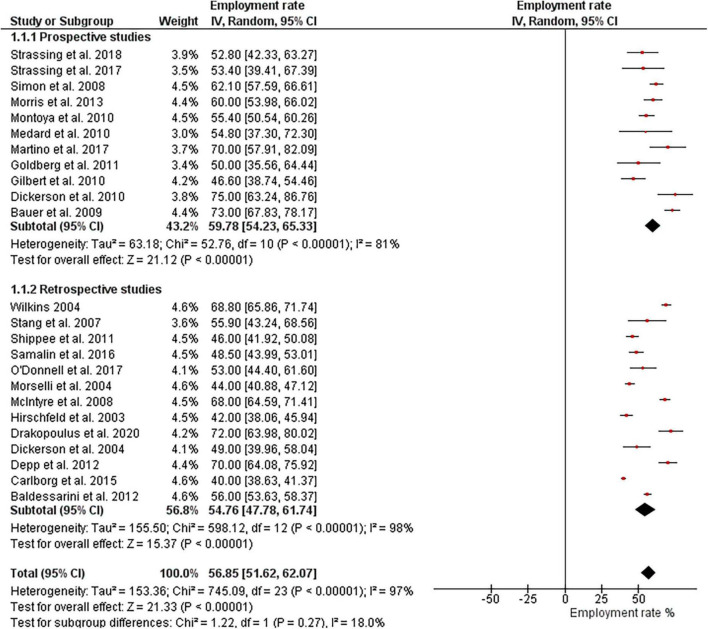
Flow chart: an overview of the study selection process.

The initial search strategy identified 5,022 abstracts. After a preliminary review of the titles and abstracts, 699 potentially relevant studies were selected. After reading the abstracts of 699 papers, 217 were qualified for full-text screening. Subsequently, full-text articles were evaluated based on the inclusion and exclusion criteria, which resulted in excluding 143 manuscripts. Overall, 74 studies met the inclusion criteria. A flowchart of the review process is shown in Figure 2.

All studies were published between the years 1977 and 2022. Interestingly, no study was identified as a result of the following keywords search: “bipolar spectrum AND employment OR unemployment OR occupation OR professional functioning.” However, out of the studies identified, the largest number concerned BD (*n* = 55), followed by MDD (*n* = 16), affective personality disorder (*n* = 11), dysthymia (*n* = 2), and affective temperament (*n* = 2). In 13 studies, more than one disorder and its relation to employment outcome were studied. Ten of included studies were ranked five stars, 27 studies–four stars, 22 studies–three stars, 12 studies–two stars, and other three studies were assessed as one star.

We included only observational (non-interventional) studies, both longitudinal and cross-sectional. The study design was prospective in 30 cases, and the mean length of follow-up was 5.8 years (ranging from 5 months to 23 years). Quantitative methods were used in all studies except for one (19), which applied qualitative design and concerned borderline personality symptoms among workers. The data from 74 papers represented a sample of 73.5 thousand individuals with at least one condition of BS disorders.

The majority of studies were conducted in the United States (*n* = 35), followed by Spain (*n* = 10), Australia (*n* = 6), Italy (*n* = 5), Canada (*n* = 4), Sweden (*n* = 3), Netherlands (*n* = 3), France (*n* = 3), United Kingdom (*n* = 3), Argentina (*n* = 2), Turkey (*n* = 2), Norway (*n* = 2), and with single representations from Switzerland, Germany, Denmark, Finland, Portugal, Russia, Israel, Colombia, Taiwan, and Japan. Three studies applied a cross-national analysis (20–22).

We distinguished two areas of concern:

– Employment outcomes–the link between a given BS disorder and employment outcomes, that is, (1) employment rate, (2) work performance (namely, presenteeism and absenteeism), and (3) work costs and earnings.
– Factors associated with employment outcomes (clinical or demographical).

The outcomes of the analysis of the first and second topics were yielded in 54 and 50 studies, respectively. Thirty studies concerned simultaneously both topics. The results are shown in Supplementary Table 1.

### Employment outcomes

#### Employment rates

Forty-two studies reported the employment and/or unemployment rate (Table 1). The majority of them (*n* = 37) concerned the BD population, seven dealt with patients with MDD, three with BPD, one included a patient with dysthymia.

**TABLE 1:** Employment and unemployment rates in bipolar spectrum disorders by country.

Country	Employment/unemployment rates by country			
	*N* (studies)	*N* (participants)	Employment rate (%) Range Mean [95%CI]* Heterogeneity test: I^2^ chi^2^ Tau^2^	Unemployment rate (%) Range
**Bipolar disorder (BD)**				
**United States** Dion et al. (100), Hirschfeld et al. (27), Dickerson et al. (65), Altshuler et al. (68), Stang et al. (26), Simon et al. (101), Gilbert et al. (73), Dickerson et al. (102), Zimmerman et al. (37), Shippee et al. (31), Goldberg and Harrow, (50), Ghaemi et al. (92), Zimmerman et al. (76), Samalin et al. (103), O’Donnell et al. (35), Strassnig et al. (104), Strassnig et al. (105)	17	3,523	42.7–75% Heterogeneity test: I^2^ = 90%, Chi^2^ = 108.21, *p* < 0.001, Tau^2^ = 106.8	21.2–58%
**Canada** Wilkins (75), Michalak et al. (34), McIntyre et al. (53)	3	1,687	36–68.8% Mean = 68.46 [95% CI 66.23, 70.69]**** Heterogeneity test: (I^2^ = 0%, Chi^2^ = 0.12, *p* = 0.73)	61.00%
**Argentina** Martino et al. (59)	1	55	70.00%	22–37%
**Australia** Waghorn et al. (24)	1	156	26.9%	73.1%
**United Kingdom, Scotland** O’Shea et al. (62), Morriss et al. (106)	2	282	60.00%	14.00%
**Spain** Martinez-Aran et al. (107), Mur et al. (70), Montoya et al. (71)	3	488	54.5%	30.00–90%
**France** Medard et al. (108), Samalin et al. (103)	2	499	48.5–54.8% Mean = 48.89 [95% CI 44.53, 53.26]**** Heterogeneity test: (I^2^ = 0%, Chi^2^ = 0.47, *p* = 0.49)	–
**Sweden** Drakopoulos et al. (69), Carlborg et al. (38)	2	5,764	39–72% Heterogeneity test: I^2^ = 98%, Chi^2^ = 59.4, *p* < 0.001, Tau^2^ = 503.3	28–61%
**Denmark** Hakulinen et al. (51)	1	2,868	–	62.00%
**Israel** Davidson et al. (23)	1	4,340	20–24%	–
**Taiwan** Chang et al. (29)	1	502	–	27.00%
**Cross-national** (France, Italy, United States Netherlands, Portugal, Spain, Canada, Switzerland, Germany, Russia, Turkey, Scotland, Sweden, Argentine) Morselli et al. (20), Bauer et al. (21), Baldessarini et al. (22)	3	2,914	27–61%	21.5–27%
**Major depressive disorder (MDD)**				
**United States** Lerner et al. (39), Goldberg and Harrow, (50), Shippee et al. (31), Zimmerman et al. (76)	4	6,810	63–88% Heterogeneity test: (I^2^ = 97%, Chi^2^ = 62.6, *p* < 0.001 Tau^2^ = 295.3)	12–37%
**Denmark** Hakulinen et al. (51)	1	23,901	–	53.00%
**Canada** McIntyre et al. (53)	1	2,323	69.00%	–
**Colombia** Uribe et al. (40)	1	107	61.00%	–
**Dysthymia**				
**United States** Lerner et al. (39)	1	59	86%	14.00%
**Borderline personality disorder (BPD)**				
**United States** Soloff and Chiapetta (109), Javaras et al. (43)	2	211	33.8–50% Mean = 42.52 [95% CI 26.69, 58.35]**** Heterogeneity test: (I^2^ = 80%, Chi^2^ = 4.9, *p* = 0.03, Tau^2^ = 104.6)	–
**Australia** Sio et al. (60)	1	60	66.7%	26.7%

*Pooled mean of employment rate calculated after excluding studies rated at 1 or 2 stars.

##### Employment rates in the bipolar disorder population

A majority of studies were performed in the United States (*n* = 20), followed by Spain (*n* = 3) and Canada (*n* = 3). It is worth noting that there were great variations in the definition of employment status. For the purpose of this study, we used a definition of “employed” as full- or part-time work or student, just as it was applied in a majority of studies.

**FIGURE 3:**
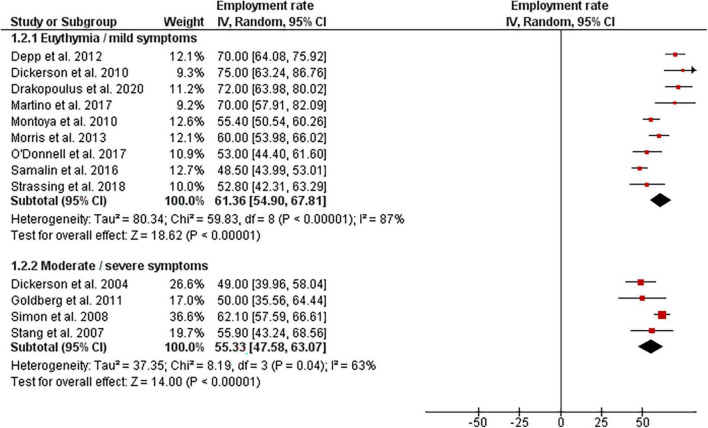
Employment rate among BD population–pooled analysis with regards to study design.

**FIGURE 4:**
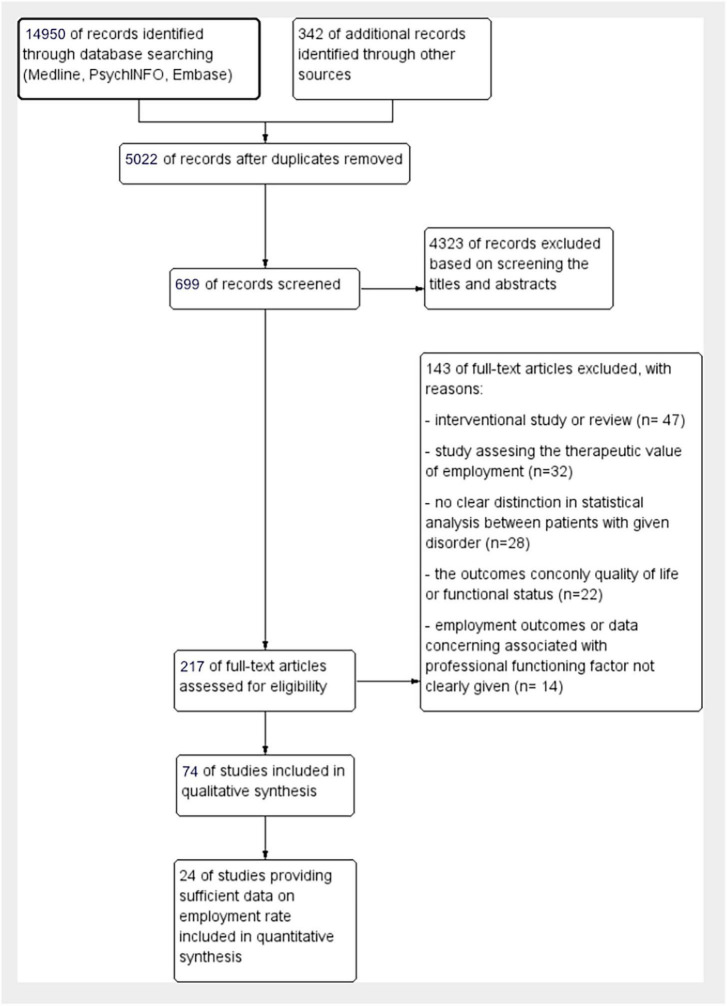
Employment rate among BD population–subgroup analysis with regard to the severity of affective symptoms.

However, there were found important discrepancies reflected in the considerable heterogeneity of analyzed studies. Specifically, we identified two significant outliers reporting very low employment rates–20 and 26.9% in Israel and Australia, respectively (23, 24). Both studies included only selected and most severely affected the population of BD individuals. Therefore, we performed a sensitivity analysis excluding all studies rated one or two stars (which also covered the two studies mentioned above) that amounted to a total of 24 studies, ranked from 3 to 5 stars. It resulted in a slight increase in the overall estimate–the global employment rate ranged from 40 to 75% (I^2^ = 97%, chi^2^ = 745 *p* < 0.001, Tau^2^ = 153.6). However, due to still large heterogeneity, the pooled global mean was not calculated. To identify other possible sources of heterogeneity, we performed subgroup analyses by study design (Figure 3) and by the severity of affective symptoms (Figure 4). A subgroup analysis by study design (prospective/retrospective) revealed the range of employment rate for prospective studies at follow-up at 46.6–75% (mean 59.8% [95% CI: 54.23, 65.33], I^2^ = 81%, Chi^2^ = 52.7, *p* < 0.001, Tau^2^ = 63.1). For retrospective studies, values were between 40 and 72%; however, there was a considerable inconsistency across studies (I^2^ = 98%, Chi^2^ = 598.1, *p* < 0.001, Tau^2^ = 155.5) (Figure 3).

As regards the severity of symptoms, it appeared that across studies involving patients with moderate to severe symptoms employment rate ranged from 49 to 62% (mean 55.3% [95% CI: 47.58, 63.07], I^2^ = 63%, Chi^2^ = 8.19, *p* = 0.04, Tau^2^ = 37.3), while in the case of euthymic patients or patients with mild severity of symptoms, the range was 48.5–75% (mean = 61.3% [95% CI:54.9, 67.81], I^2^ = 87%, Chi^2^ = 59.8, *p* < 0.001, Tau^2^ = 80.3) (Figure 4).

Visual evaluation of all funnel plots showed a symmetrical distribution, thus indicating the absence of publication bias.

##### Employment rates in individuals with major depressive disorder, dysthymia, and borderline personality disorder

The values for BPD calculated on the basis of three higher quality studies (four stars) were comparable or even lower than in BD individuals and ranged from 33.8 to 66.7% (mean = 50.1% [95% CI 33.70, 66.60], I^2^ = 86%, Chi^2^ = 14.7, *p* < 0.001, Tau^2^ = 181.6). The values for individuals with MDD obtained from five studies (scored 3–5 stars) oscillated between 61 and 88% (I^2^ = 96%, Chi^2^ = 94.2, *p* < 0.001, Tau^2^ = 36.8). The employment rate for individuals with dysthymia was assessed in only one study (five stars) at 86%.

#### Work performance

Another problem related to the employment of people with affective disorders is their productivity at work, which is attributable to absenteeism and presenteeism (25). Twenty-six studies provided data concerning work performance, of which 17 concerned BD population, 7 MDD population, 4 borderline personality symptoms and 2 patients with dysthymia (Supplementary Table 1). Identified studies varied greatly in terms of the tools used to evaluate this issue and time frames applied to assessing absenteeism. Therefore, pooled analysis was problematic.

All the studies mentioned above showed that work under-performance was closely related to the presence of BS disorders. In particular, BD appeared to have a serious negative impact on the careers of affected individuals (26, 27, 28). However, Chang et al. (29) (five stars) in the longitudinal study found out that this negative effect was pronounced a year before the diagnosis of BD, with a gradually decreasing risk over the subsequent 2 years, and a comparable one to control outcomes from the third year onward. According to one of the surveys performed by Stang et al. (26), 41% of BD individuals reported fearing the loss of their current job due to their emotional state. Indeed, a greater incidence of being fired and a decreased likelihood of employment in this population could be compared to controls found in several prospective and retrospective studies scored at 3–5 stars (30–33). The authors of two other studies pointed out main topics related to worse professional functioning of BD individuals such as lack of continuity in work history, interpersonal problems at work, inadequate illness management strategies in the workplace, stigma, and exclusion at work (34, 35). As regards work productivity in the BD population, four studies (all ranked at four stars) reported that long-term absences from work were significantly more frequent in this population than in controls (31, 36–38).

One study by Lerner et al. (39) (five stars) assessed that individuals with MDD had significantly greater presenteeism and absenteeism as compared to controls. Uribe et al. (40) (three stars) reported that absenteeism concerned 70% and presenteeism among 99% of patients with MDD. Also, in this population, there were more job turnovers than in healthy controls (39).

As it was assessed in two studies (four and five stars), individuals with dysthymia also appeared to have less stable work histories and a greater frequency of significant problems at work than controls (39, 41). Appositely, significantly greater presenteeism and absenteeism were noted in this population than in controls (39). However, Adler et al. (41) revealed that absenteeism was similar among individuals with dysthymia and healthy controls, while presenteeism was, indeed, significantly greater in individuals with dysthymia.

Moving to the severity of the spectrum axis, four papers (two–two stars, one–three stars, and one–four stars) assessed the impact of borderline personality symptoms on work performance. Individuals with BPD found employment circumstances stressful and difficult to cope with (42) and occupational impairment was observed in this group (43). The presence of borderline personality symptoms was associated with a greater total work loss days and greater job insecurity if compared to controls (44) or losing a job on purpose (45).

Regarding a comparative analysis of BS disorders, three studies juxtaposed occupational stability between patients with BD-I and BD-II, which provided ambiguous results. Arvilommi et al. (46) (four stars) over a 6-year follow-up period found that patients with BD-I were granted a disability pension more often than patients with BD-II. Similarly, Dell’Osso et al. (47) (four stars) found more favorable outcomes for BD-II individuals, while Ruggero et al. (48) (four stars) concluded that BD-II was associated with serious work impairment that was more similar to BD-I than different from it. We found that there is a lack of more studies analyzing BD-I and BD-II patient groups comparatively. In contrast to papers analyzing patients with BD-I, which are numerous, there is a lack of papers concerning only patients with BD-II. Thus, a more in-depth comparison of the two types of BD was not possible.

Six other studies (two–three stars and four–four stars) provided evidence that individuals with MDD had consistently better overall work functioning as compared to BD (49–51), also in terms of work productivity (31, 48, 52). While comparing MDD and dysthymia, presenteeism and absenteeism were notably more pronounced in the group of MDD individuals; moreover, significant problems at work were more prevalent in this population as well (39).

#### Work cost and earnings

Work costs and salaries of individuals with BS disorders were assessed in 11 studies (Supplementary Table 1). Eight of them concerned BD population, six-patients with MDD and one investigated the link between dysthymia and output lost. No study concerning any other disorder apart from BS was identified.

Regarding BD and salaries, four studies (two–three stars and two–four stars) reported lower annual income of patients with BD if compared to the general population, despite similar education levels (30, 38, 51, 53). This difference in income was estimated in the study by Hakulinen et al. (51) at around 36% less for BD individuals and 51% less for depressive patients if compared to individuals without affective disorders.

As regards the work cost of employees with affective disorders, other four studies (one–three stars and three–four stars) evaluated this issue. Gardner et al. (36) assessed that costs of employees with BD were three times higher than those without this diagnosis. Similarly, Shippee et al. (31) estimated that individuals with BD and MDD had higher work-related costs than controls and this difference was more pronounced in patients with BD. Kessler et al. (52) also calculated annual capital loss per ill person at $9.6 and $4.4 thousand for patients with BD and MDD, respectively. One study also estimated output lost due to dysthymia ($2.8 thousand) which was a significantly higher value in comparison to healthy controls ($1.2 thousand) (41).

### Factors influencing employment outcomes

A total of 50 studies reported factors associated with professional functioning. A majority of them (*n* = 41) concerned the BD population, eight dealt with patients with MDD, six with BPD, and two with affective temperament (Supplementary Table 1; Table 2).

**TABLE 2:** Factors associated with employment outcomes in individuals with BS disorders.

Factors significantly associated with employment outcomes	Studies reporting on given variable % (n/N)–percent, (n-number of studies where variable was significant/N-number of all studies evaluating given variable)				
	Bipolar disorder (BD)	Major depressive disorder (MDD)	Borderline personality disorder (BPD)	Dysthymia	Affective temperament
**Sociodemographical factors**					
**Age** Waghorn et al. (24); Rosa et al. (110), Dickerson et al. (102); Zimmerman et al. (37); Goldberg and Harrow, (50); Grande et al. (111); Caruana et al. (78); Arvilommi et al. (46)	71.4% (5/7)	50% (1/2)	100% (1/1)		
**Age of onset of the illness** Dickerson et al. (65); Baldessarini et al. (22)	50% (1/2)				
**Gender** Sansone et al. (55); Witt et al. (91); Buoli et al. (54)	50% (1/2)		100% (1/1)		
**Education** Glibert et al. (112); Caruana et al. (78); Hakulinen et al. (51)	100% (3/3)	100% (2/2)	100% (1/1)		
**Cognitive performance**	82.3% (14/17)	100% (2/2)			
Dickerson et al. (65); Altshuler et al. (68); Kaya et al. (113); Martinez-Aran et al. (107); Mur et al. (70); Gilbert et al. (112); Burdick et al. (66); Dickerson et al. (102); O’Shea et al. (62); Depp et al. (114); Schoeyen et al. (67); Ryan et al. (115); Lawrence et al. (82); Boland et al. (33); Strassnig et al. (104); Sole et al. (64); Drakopoulos et al. (69)					
**Symptoms and course of the illness**					
**Number of hospitalizations**	85.7% (6/7)	100% (1/1)			
**Number/severity of depressive episodes**	77.8% (14/18)	100% (2/2)			
**Number/severity of manic episodes**	40% (4/10)				
**Number/severity of other symptoms**	100% (2/2)		75% (3/4)		
**Remission/recovery rates**	100% (2/2)				
Dickerson et al. (65); Wilkins (75); Kessler et al. (52);Waghorn et al. (24); Simon et al. (101); Bauer et al. (21); Mur et al. (70); Rosa et al. (110); Burdick et al. (66); Zimmerman et al. (37); Dickerson et al. (102); Reed et al. (116); Sio et al. (60); Goldberg and Harrow, (50); Haro et al. (117); Depp et al. (114); Grande et al. (111); Morriss et al. (106); Schoeyen et al. (67); Ryan et al. (115); Boland et al. (33); Martino et al. (59); O’Donnell et al. (35); Strassnig et al. (104); Miller et al. (118), Juurlink et al. (19); Hakulinen et al. (51); Drakopoulos et al. (69); Soloff and Chiapatta (109); Woodhead et al. (118); Arvilommi et al. (46)					
**Comorbid psychiatric disorders**					
**Substance abuse/dependence** Waghorn et al. (24); Dickerson et al. (102); Zimmerman et al. (37); Soloff and Chiapatta (109)	75% (3/4)	0% (0/1)	100% (1/1)		
**Personality disorders** Medard et al. (108); Grande et al. (111); Zimmerman et al. (76); Arvilommi et al. (46)	100% (4/4)	100% (1/1)			
**ADHD** Landaas et al. (56)					100% (1/1)
**Anxiety** Zimmerman et al. (37); Soloff and Chiapatta (109); Arvilommi et al. (46)	100% (2/2)		100% (1/1)		
**Depressive symptoms** Soloff and Chiapatta (109); Tei-Tominaga et al. (57); Arvilommi et al. (46)	100% (1/1)		100% (1/1)		100% (1/1)
**Post-traumatic stress disorder** Arvilommi et al. (46)	100% (1/1)				
S**ubsyndromal/Residual symptoms**					
**Subsyndromal depressive symptoms** Kaya et al. (113); Bauer et al. (21); Mur et al. (70); Montoya et al. (71); Goldberg Harrow, (50); Samalin et al. (103); Sole et al. (64)	100% (8/8)	0% (0/1)			
**Subsyndromal manic symptoms** Mur et al. (70); Montoya et al. (71); Samalin et al. (103); Montoya et al. (71)	100% (4/4)				

#### Sociodemographic factors

Fourteen studies provided data on sociodemographic factors. We identified four factors significantly related to employment outcomes: age, age of the onset of the disorder, gender, and education. The most consistent findings came from eight studies (one–five stars, four–four stars, two–three stars, and one–one star) reporting poorer occupational functioning among older individuals with BD, MDD, and BPD (Table 2). The impact of gender on professional functioning was confirmed in 50% (1/2) studies concerning BD–female gender was less frequently associated with employment in the study of Buoli et al. (54). Similar findings were provided by the study on individuals who met the criteria for BPD–employment disability was found only among women (55). The years of education also appeared to be significantly associated with employment trajectory among individuals with BS disorders, which was confirmed in 100% of studies (3/3) (Table 2).

#### Comorbidity with other mental disorders

Ten studies (two–five stars, four–four stars, two–three stars, one–three stars, and one–one star) provided evidence, indicating that patients with BS disorders had worse indicators of occupational performance in the case of comorbidity with other mental disorders (Table 2). Work under-performance among BD individuals was associated with increased rates of anxiety in two studies, post-traumatic stress disorder (PTSD) in one study, and alcohol abuse or dependence in 75% (3/4) of other studies. Particularly, unfavorable employment outcomes were noted in studies estimating comorbidity of BD (100%, 4/4) or MDD (100%, 1/1) with personality disorders. Another comorbidity was described in the study by Landaas et al. (56) (three stars), and cyclothymic temperament was highly prevalent in adults with ADHD and strongly associated with lower occupational achievements, as well as with increased comorbidity, in particular with BD. As regards cyclothymic and anxious temperament, it was proved to be a high-risk factor for depressive symptoms (57) (two stars). This study was performed in a group of workers in their 20–40s in Japan, where immature-type depression (frequently classified as belonging to the bipolar spectrum) is commonly observed and may be triggered by work-related stressors. The clinical picture includes dependency and aggression related to patients’ immature personalities; additionally, cyclothymic temperament is also highly prevalent in this condition (58).

#### Symptoms and course of the illness

The number of hospitalizations, remission rates, and affective symptoms severity was found to be associated with work impairment among individuals with BD, MDD, as well as BPD (Supplementary Table 1; Table 2) in 31 mainly high-quality studies (six–five stars, 15–four stars, seven–three stars, one–two stars, and two–one star). In particular, a higher number or severity of depressive episodes was especially associated with work impairment and unemployment as was shown in 77.8% (14/18) and 100% (2/2) studies among patients with BD and MDD, respectively (Table 2). Furthermore, by comparing the impact of BD and MDD on work performance, Kessler et al. (52) found less favorable outcomes related to more severe and persistent depressive episodes among BD individuals. For manic symptoms, the relationship was more blurred as it was confirmed in only 40% (4/10). Also, a higher number of lifetime hospitalizations was related to occupational status–such an association was found in 85.7% (6/7) and 100% (1/1) studies among BD and MDD individuals, respectively. Moreover, one long-term study provided evidence that over time occupational outcomes tended to remain stable or even slightly improved (59).

As regards BPD, symptoms related to the clinical characteristics of this condition such as difficulty in posing personal boundaries or regulating emotions were also associated with professional functioning (19). Additionally, those who experienced more severe emptiness, impulsivity, and self-harm had worse outcomes (60, 61).

#### Cognitive performance

Seventeen studies focused on the evaluation of often prolonged impaired disturbances in cognitive functions (one–five stars, six–four stars, five–three stars, and five–two stars). The majority of them, 82.3% (14/17) and 100% (2/2), confirmed a link between cognitive performance and work impairment in the population of BD and MDD individuals, respectively (Supplementary Table 1; Table 2). Employment outcomes were associated with various cognitive functions such as attention (62, 63), processing speed (64), immediate verbal memory (65), or verbal learning (66), while IQ was unrelated to these measures (67). However, the most highlighted aspect was the role of executive functions perceived as a powerful predictor of occupational status and work adjustment in patients with BD (68, 69).

#### Subsyndromal or residual symptoms

As syndromal remission in affective disorders was not always accompanied by normal functioning (70, 71), the impact of subthreshold or residual symptoms was noticed. The presence of subsyndromal symptoms, which is referred to the second dimension of BS definition, turned out to be one of the possible explanations.

We identified seven studies that assessed the impact of subsyndromal or residual affective symptoms on employment outcomes (one–five stars, three–four stars, one–three stars, and two–two stars) (Supplementary Table 1; Table 2). Interestingly, all identified studies among BD individuals confirmed a significant association between subsyndromal depressive symptoms and employment outcomes. For example, Bauer et al. (21) found that disabled patients suffered from subsyndromal depression two times as frequently as those with full-time employment. The analogous relation of subsyndromal manic symptoms was also found in all identified studies; however, this evidence came from half the number of studies if compared to subsyndromal depressive symptoms (*n* = 4).

## Discussion

Treatment outcomes in affective disorders have been traditionally determined by the assessment of clinical characteristics such as recurrence rates or syndromal remission. However, it has been proven that employment plays a central role in the lives and identities of individuals with mental disorders and returning to work is an integral part of their recovery (72). Furthermore, apart from fully symptomatic affective disorders clearly disturbing an individual’s ability to work (17), the impact on work performance of softer or subsyndromal affective symptoms remains unclear. To the best of our knowledge, this is the first systematic review concerning this topic, bearing in mind such a fairly broad spectrum of conditions.

### Employment outcomes

Occupational difficulties that have emerged from the review of literature include difficulties in maintaining employment, reduced work productivity, lower earnings, and higher labor cost. The available studies describe mainly the effect of BD on occupation with only a few studies related to MDD, dysthymia, and BPD. Based on the literature review mentioned above, all BS disorders appeared to have a negative impact on the employment rate. The employment rates were lowest among BD individuals–40–75% and even lower among individuals with BPD–33.8–66.7%. In general, the estimates for BD are similar to those reported in other reviews concerning the BD population–61–75% (73) and 40–60% (17). Individuals with MDD (61–88%) and dysthymia (86%) appeared to have higher percentage points of employment rates. Importantly, in the case of comorbidity of two or more BS disorders (in particular personality disorders), employment rates appeared to be lower than for a single disorder. This is in line with other reviews on comorbid personality disorders in individuals with BD, which revealed poorer functional outcomes in such comorbidity (74).

Understandably, employment rates in different countries vary greatly due to differences in socioeconomic background and healthcare systems. However, according to the Eurostat data, (4) the employment rate in the European and United States general population was between 62 and 66% and 66 and 74%, respectively. Thus, BD and BPD appeared to have worse rates if compared to the statistics mentioned above. These findings are also supported by other studies, which juxtaposed BD individuals with healthy controls–in comparison to controls, approximately 10–30% fewer BD individuals were available for work (31, 38, 75). Similarly, values for BPD were far below estimates in controls (43). It seems that also outcomes for individuals with MDD could be below the general population as was shown in two studies (31, 39).

However, the problem with the employment of individuals with affective disorders appeared to be much more complex than just lower employment rates. We have also concluded that employees with affective disorders have great problems related to the overall work performance, greater absenteeism and presenteeism, and lower income (25, 36). Those findings apply to both patients with BD as well as individuals with MDD, dysthymia, and BPD (39, 41, 43). It is worth emphasizing that the outcomes of this review pointed to consistently better work performance, including higher employment rates, in MDD than BD populations.

Interestingly, although employment rates in individuals with dysthymia were found to be similar in the general population, it was possible to observe lower work productivity expressed especially in presenteeism (41). Nevertheless, under-performance for dysthymia was less pronounced than in the MDD population. Finally, the problem of occupational impairments was also reviewed in individuals with BPD. The results of this study suggest that professional functioning in this population is similar to BD. In several other studies, the likelihood of vocational disengagement also did not differ between individuals with BD and BPD (76–78).

### Factors influencing employment outcomes

The identification of risk factors of occupational functioning in BS seems to be crucial for preventing retirement and premature occupational disability. It is noteworthy that the studies identified in the present review mainly evaluated this issue among the BD population, with only single representations for MDD, dysthymia, BPD, and affective temperament. Despite some inconsistencies, we identified five groups of factors with the strongest evidence for association with employment outcomes: sociodemographic (in particular age and gained education), symptoms, and course of the illness (number of hospitalizations, number/severity of symptoms, mainly depressive), cognitive functions, comorbidity (with substance abuse, personality disorders, anxiety, ADHD, PTSD, and depressive symptoms), and persistent subsyndromal symptoms (in particular depressive). Taking into account, variables that were evaluated in at least eight studies, the highest signal strength in BD individuals concerned cognitive performance, the number/severity of depressive symptoms, and the presence of subsyndromal depressive symptoms. Kessler et al. (52) assessed that subthreshold depressive symptoms were unrecognized causes of long-term negative work outcomes considerably more disadvantageous in the BD population in comparison to MDD. Subthreshold depressive symptoms are also present in dysthymia, which appears to be an unrecognized cause of work impairment with even more long-term negative consequences (41). Thus, the effort focused beyond syndromal remission and targeted subsyndromal symptoms, with functional recovery appearing to be of great importance (71). A large number of analyzed studies have also focused on the evaluation of disturbances in cognitive functions as a predictor of professional performance. We conclude that the evidence clearly indicates that this is an important variable related to employment outcomes as this has been confirmed in the majority of studies concerning BD and MDD populations. This is in line with other systematic reviews on this topic among BD individuals (79, 80), as well as with the meta-analysis (81). Understandably, some of the factors such as cognitive functions and depressive symptoms might be intercorrelated. This problem was raised in the study by Lawrence et al. (82). Authors of this study have proved that severe depressive symptoms are correlated with cognitive dysfunctions. Regardless of the inconsistencies mentioned above, the issues of better management of depressive symptoms as well as cognitive difficulties seem to be very important in BS disorders.

In this study, we used the BS model that was proposed by Angst (10). However, it should be highlighted that the concept of the BS itself is still being discussed. In general, the BS concept can be approached in two different ways: (1) manic–depressive spectrum–continuum between bipolar and unipolar, and (2) restricted to bipolar disorder with a continuum between full-blown illness (BD-I) through milder illness to temperament traits (83). In particular, the inclusion of MDD and dysthymia in BS disorders can be controversial. It should be emphasized that, in recent years, important data have been provided by genetic studies (84–86). Coleman et al. (86) analyzed subtypes of MDD and BD and provided evidence for a genetic mood disorders spectrum. The authors revealed that BD-II correlates strongly with recurrent and single-episode MDD. The results of this study suggest a spectrum of genetic links between MDD and BD with BD-II bridging the gap between the two disorders. Similarly, the inclusion of BPD in the BS in Angst’s concept may be controversial. It is worth mentioning that other authors also consider BPD as a part of a spectrum of affective disorders (87, 88). They point to the high co-occurrence of both disorders, positive family histories and shared symptoms. Certainly, mood disorders are very common among patients with BPD (89). A study by Sjåstad et al. (90) found that patients with BPD have a significantly higher risk of BD (by 66%) compared to an aggregated group of other personality disorders. More light can also be shed here by the genetic study showing a genetic overlap of BPD with BD and MDD (91). Yet another approach to the BS concept is presented by Ghaemi et al. (92). The authors described BS disorder as a condition that clinically ranked between unipolar and bipolar disorder but failed to meet the criteria for any of them. Moreover, according to Akiskal and Pinto (11), the definition of BS covered all forms of affective disorders that showed the features of bipolarity, including affective temperaments. It is of note that temperaments are perceived rather as vulnerability factors that could modify the course of the illness. Specifically, cyclothymic, depressive, and irritable temperaments were found to predict poor response to treatment and suicidal behavior in BD, whereas hyperthymic temperament appeared to be protective (93, 94). Unfortunately, we could identify only two studies concerning affective temperament in the context of professional functioning; hence, any firm conclusions could not be drawn. It is worth noting, however, that cyclothymic temperament was highly prevalent in adults with ADHD and associated with increased comorbidity with BD. The genetic study also indicates an overlap between ADHD and BD (95).

It is also worth mentioning that patients with BD-II with cyclothymic temperament were found to be often misdiagnosed as having BPD (96, 97). We conclude that misdiagnosis of BPD/BD in some of the studies (in particular where the diagnosis was not clinically based) may at least partly explain poor employment outcomes seen in the BPD population.

## Limitations

In this review, the conclusions are based on a relatively small number of studies concerning disorders other than BD, thus being subject to change by adding further studies. Furthermore, a large number of studies involved small samples and were cross-sectional limiting conclusions regarding causal directions associated with employment factors. Furthermore, there was little geographical spread within studies as they were performed mainly in the United States. Since differences related to healthcare and welfare systems across countries exist, we cannot state for sure that under-reporting from other parts of the world would not impact the outcomes of this study. We have also included only studies written in English. The majority of studies did not examine employment rates with the data being collected for other purposes; hence, this issue could be in general under-reported. Due to the differences in outcome measures mainly as well as varying study populations, outcomes could not be pooled and meta-analyzed. We only reported pooled employment rates; however, in most cases, there was considerable heterogeneity. Moreover, in observational studies, a substantial inconsistency across studies is almost always expected (98).

## Conclusion

The results of this review have shown that disorders included in the BS have a negative impact on occupational status, work performance, work cost, and earnings of individuals. It appears that BD has the greatest unfavorable impact on employment out of all BS disorders. Several lines of evidence also indicate that BPD may have a comparable disruptive effect to that of BD. Similarly, work under-performance was noted among individuals with MDD and dysthymia, although it was less pronounced than in BD. It is of note that data regarding other disorders included in BS such as affective personality disorders, dysthymia, cyclothymia, or affective temperaments are rather few. Further research in this area would be particularly important as it has appeared that also subthreshold symptoms have a detrimental effect on professional functioning. There is a clear need for studies, preferably longitudinal, focused on other than classic forms of affective disorders and their impact on occupation, performed in different socioeconomic backgrounds. The outcomes such as performance at work as well as factors associated with occupational outcomes in individuals with BS disorders other than BD are significantly understudied. Although a recent review of measurement tools in the BD population indicated a tendency toward uniformity in applied functional outcome measures (99), a greater uniformity would be highly desirable in studies on other BS disorders.

## Data availability statement

The original contributions presented in this study are included in the article/Supplementary material, further inquiries can be directed to the corresponding author.

## Author contributions

AA-W generated the idea. MD, PJ, and AA-W designed the study, performed the systematic search and publication review, performed the data extraction, and interpreted the results. MD and PJ wrote the first drafts of the manuscript. MD wrote the final version of the manuscript. ŁŚ, PB, MK, and HS-J critically reviewed the manuscript. All authors contributed to the article and approved the submitted version.

## Supplementary material

Supplementary Table 1 Characteristics of studies investigating employment outcomes (employment rate, work productivity, labor costs, earnings, work performance) and factors associated with professional functioning in individuals with bipolar spectrum disorders.
