# Supplementary material for: The impact of bipolar spectrum disorders on professional functioning: A systematic review
Source: Front Psychiatry. 2022 Aug 24;13:951008. doi: 10.3389/fpsyt.2022.951008 (PMC9448890; doi:10.3389/fpsyt.2022.951008)
Supplement: Supplementary Table 1 — Characteristics of studies investigating employment outcomes (employment rate, work productivity, labor costs, earnings, work performance) and factors associated with professional functioning in individuals with bipolar spectrum disorders. [file Table_1.pdf]

Characteristics of studies investigating employment outcomes (employment rate, work productivity, labour costs, earnings, work performance) and factors associated with professional functioning in individuals with bipolar spectrum disorders.

Note: BD-bipolar disorder, BD-I – bipolar disorder type 1, BD-II- bipolar disorder type 2, MDD- major depressive disorder, BPD-borderline personality disorder

No	Author Year Country	Study group	Study design	Employment outcomes	Factors associated with professional functioning	Quality assessment
**1**	Adler et al. 2004 (40)<br><br>USA	69 individuals with dysthymia<br>Mean age: 40<br>F/M (%): 81/19<br>Depressive symptoms severity: 9.7 (based on PHQ-9)<br><br>175-controls<br>Mean age: 41<br>F/M (%): 72/28<br>Depressive symptoms severity: 2.8 (based on PHQ-9)	Prospective (18 months)	**Work performance:**<br>-Patients with dysthymia, compared with controls, had: more frequent occupational and employer changes ($p<0.0001$), a greater frequency of significant problems at work, health issues had caused them to move from full-time to part-time employment ($p<0.0003$), to take lower paying jobs ($p<0.003$) and had a history of stopping work – for >30 days or longer due to physical or emotional problems ($p<0.003$)<br>**Absenteeism:**<br>-Absenteeism was similar in dysthymia and control group (10.8% vs. 7.5%, respectively, $p<0.25$)<br>**Presenteeism:**<br>-Presenteeism was significantly greater among individuals with dysthymia as compared to controls (6.3% vs. 2.8%, $p<0.0001$) (according to WLQ Productivity Loss Index)<br>**Work costs:**<br>-The output lost was significant - dysthymia vs. control, \$2890 vs. \$1292 ($p<0.01$)		4 (b,c,d,e)

**2**	Altshuler et al. 2007 (65)<br><br>USA	213 patients with BD (types I and II) admitted to hospital due to: depression (47%), mania (29%), mixed state (23%)<br>Mean age: 43.3<br>F/M (%): 9/91	Retrospective	**Employment status:**<br>-Employed (full- or part-time) – 42.7% (n=91)<br>-Unemployed – 57.3% (n=122)	**Cognitive performance:**<br>-Unemployed group showed greater executive functions impairment (p=0.006)	2 (a,d)
**3**	Arvilommi et al. 2022 (117)<br><br>Finland	152 patients with BD (68 - types I and 84 - II)	Prospective (6 years)	**Work performance:**<br>55,5% of the patients were granted a disability pension	**Sociodemographical factors, Symptoms / course of the illness,**<br>-Older age; type I BD; comorbidity with generalized anxiety disorder, post-traumatic stress disorder or avoidant personality disorder; duration of time with depressive or mixed symptoms predicted disability pensions	4 (a,b,d,e)
**4**	Baldessarini et al.<br>2012 (22)<br><br>Italy, Spain, Argentine, US, Turkey, Switzerland	1,665 patients with BD type 1 (BD- I)<br><br>Mean age: 40.8<br>F/M (%): 52/48	Retrospective	**Employment status:**<br>- Employed – 55.9%	**Sociodemographical factors:**<br>-Lower employment rate was associated with younger, especially childhood onset of disease as compared to adolescent (12-18) and adult (19-55) (37.%% vs 62% vs 72%, respectively)	3 (a,b,d)
**5**	Bauer et al. 2009 (21)<br><br>USA, Canada, Germany	281 patients with BD divided into groups according to employment status: disabled (n =75), full-time employee /full-time student (n =135), other (n =71)<br><br>Mean age: 38<br>F/M (%): 71/29<br>Severity of symptoms was based on self-reported mood-ratings	Prospective (5 months)	**Employment status:**<br>-Full time employment - 48%<br>-Other (including part-time employment) – 25%<br>-Disabled – 27%	**Symptoms / course of the illness, subsyndromal symptoms:**<br>-Frequent subsyndromal symptoms, especially depressive, may preclude full-time responsibilities and contribute to disability in BD<br>-No differences in the percent of days spent in depressed or manic episodes, or in the severity of symptoms between disabled and full-time groups<br>- Disabled patients spent about twice as many days with subsyndromal symptoms of depression as those in	4 (a,b,c,d)

		previously validated with clinician ratings on HAMD and YMRS			the full-time group (29 vs. 15%, p < 0.001)<br>- Mean percent of days in any episode plus the mean percent of days with subsyndromal symptoms was significantly larger when comparing the disabled with the full-time group (44 vs. 29%, p<0.002)	
**6**	Boland et al. 2015 (32)<br><br>USA	24 euthymic BD patients<br>Mean age: 32.6<br>F/M (%): 62/38<br>Symptoms severity: 5.08 (based on BDI)<br><br>24 healthy controls	Cross-sectional, retrospective	**Work performance:**<br>- BD participants experienced significantly greater months of unemployment as compared to controls (31.5 vs 7.71, p=0.04) and greater incidence of being fired than controls (1.13 vs 0.08, p<0.001)	**Cognitive performance, symptoms / course of the illness:**<br>- BD euthymic participants experienced significantly poorer cognitive functioning (executive functions)<br>- Poor sleep and cognitive functioning were associated with poor work performance in BD participants, but not controls	3 (b,c,d)
**7**	Burnett-Zeigler et al. 2012 (31)<br>US	22,407 of respondents in National Epidemiological Survey on alcohol related conditions (3%- BD, 7.5%-MDD)	Longitudinal (4 years)	**Work performance:**<br>**-**Diagnoses of MDD or BD predicted a decreased likelihood of employment 4 years later		4 (a,c,d,e)
**8**	Buoli et al. 2019 (52)<br><br>Italy	1674 patients with BD<br>F/M (%): 57/43	Retrospective multicenter		**Sociodemographic factors:**<br>- Female gender was less frequently associated with employment (p< 0.01) and more frequently associated with a number of lifetime major depressive episodes as compared to male (p< 0.01)	3 (a,b,d)
**9**	Burdick et al. 2010 (63)<br><br>USA	33 patients with BD<br><br>Mean age: 40.2<br>F/M (%): 46/54<br>Symptoms severity: 15 % - depressive episode, 21% - manic episode	Longitudinal (15 years)		**Symptoms, course of the illness, cognitive performance:**<br>- Three variables were significant independent predictors of work functioning: verbal learning performance (p= 0.02); recent depression (p= 0.008); number of lifetime hospitalisations (p= 0.003)	4 (b,c,d,e)

					-Each of above factors contribute independently to poorer occupational outcome	
**10**	Carlborg et al. 2015 (37)<br><br>Sweden	9.4 million residents nationwide registry study in Sweden (4629 – BD in 2006, 5644-BD in 2009)<br><br>Mean age: 40.3<br>F/M (%): 64/36	Retrospective	**Employment status among BD individuals:**<br>- Working – 40.7% (2006), 39% (2009)<br>- Not working – 59.3 (2006), 61% (2009)<br>- Approximately 30% fewer patients with BD were available for work as compared to general population<br>**Work performance:**<br>**Absenteeism:**<br>-Among the 40% employed patients with BD, 64% reported sick leave<br>-Long- term sick leave (episodes of ≥100 days and ≥300 days) were common in BD individuals - 32% and 14%, respectively<br>**Earnings:**<br>-Despite similar education levels, disposable income was lower among BD patients compared to the general population		4 (a,b,c,d)
**11**	Caruana et al. 2019 (75)<br><br>Australia	Individuals with:<br>mood disorders (n = 52)<br>psychosis (n=50)<br>BPD (n= 43)<br><br>Age range: 15-25<br>F/M (%): 60/40	Cross-sectional cohort		**Sociodemographic factors:**<br>- Being not in education, employment or training were associated with not having commenced tertiary education (p = 0.002) and with being older (p = 0.004)<br>- The likelihood of vocational disengagement did not differ across groups	3 (b,c,d)
**12**	Chang et al. 2016 (28)<br><br>Taiwan	502 patients with BD<br>2008 healthy controls (data from the National Health Insurance Research Database of	Longitudinal	**Employment status:**<br>- The probability of changing a status to unemployed was significantly higher in patients with BD than in controls over time (27% vs 14%, respectively, p <0 .		5 (a,b,c,d,e)

		Taiwan)<br><br>Mean age: 30<br>F/M (%): 47/53		0001), even before the incidence of BD<br>**Work performance:**<br>- Risks of occupational deterioration in patients with BD were greater in the year before incidence and in the following year, with gradually decreasing risks over the subsequent 2 years, and comparable to controls from the third year onward		
**13**	Davidson et al. 2016 (23)<br><br>Israel	35 673 participants, including patients with BD (n=4340), schizophrenia or other non affective psychotic disorders<br>Mean age: 48	Cross-sectional Retrospective	**Employment status:**<br>- 24% of BD patients with 1 admission and 20% with multiple admissions to hospital were employed (in general population 56%)<br>**Earnings:**<br>-The percentage of BD patients earning minimum wage or above was low (ranging from 9% to 25%) and this decline in ability to work begins even years before the first admission		2 (a,c)
**14**	Dell'Osso et al. 2017 (45)<br><br>Italy	141- BD-I euthymic<br>76 - BD-II euthymic	Retrospective	**Work performance:**<br>-BD-II compared with BD-I patients had significantly more favourable occupational stability, but significantly longer duration of untreated illnessa and more frequent lifetime anxiety disorders comorbidity		4 (a,b,c,d)
**15**	Depp et al. 2012 (99)<br><br>USA	229 outpatients with BD-I<br><br>Mean age:<br>Employed – 44.3<br>Unemployed – 49.4<br><br>F/M (%):<br>Employed – 47/53<br>Unemployed – 55/45<br><br>Symptoms severity:	Cross-sectional Retrospective	**Employment status:**<br>-Employed - 70% (n=160)<br>-Unemployed - 30% (n=69)	**Symptoms, course of the illness, cognitive performance:**<br>-Neurocognitive ability was the strongest predictor of employment (p=0.006)<br>- Depressive symptoms severity did not differ between unemployed and employed patients<br>-Depressive symptom severity was the only variable significantly related to hours worked among those who were employed (r=-0.326, p<0.001)	3 (a,c,d)

		Employed: 8.56 (based on BDI)<br>Unemployed:12.6 (based on BDI)				
**16**	Dickerson et al.<br>2004 (62)<br><br>USA	117 persons with BD<br>Mean age: 41.4<br>F/M (%): 70/30<br><br>Symptoms severity:<br>Employed – BPRS: 32.8, YMRS:3<br>Part-time employed – BPRS: 36.1, YMRS:6.2<br>Unemployed - BPRS: 38.9, YMRS:6.8	Cross-sectional	**Employment status:**<br>-Employed – 27.3% (n=32)<br>-Part-time employed – 21.4% (n=25)<br>-Unemployed – 51.3% (n=60)	**Symptoms, course of the illness, cognitive performance, sociodemographic factors:**<br>-Employment status was significantly associated with cognitive performance, (especially immediate verbal memory), total symptoms severity, history of psychiatric hospitalisation and maternal education<br>-No association was found between employment status and history of psychotic symptoms, number of years of education, or age at onset of illness	4 (b,c,d,e)
**17**	Dickerson et al.<br>2010 (98)<br><br>USA	52 patients with BD-I (manic, mixed, or depressed type), BD-II or otherwise not specified<br><br>Mean age: 30.5<br>F/M (%): 85/15<br>Symptoms severity at follow-up: HDRS: 11.1, BPRS: 29.7, YMRS: 2.6	Longitudinal (6 months)	**Employment status:**<br>-Employed - 75% (n=39)<br>-Unemployed (or on sick leave) – 25% (n=13)	**Symptoms, course of the illness, cognitive performance, comorbid disorders, sociodemographic factors:**<br>-Full-time occupational status at follow-up was predicted by the absence of baseline substance abuse – 72% (n=18) persons without substance abuse were full- time workers or students as compared to only 38% (n=10) with substance use (p=0.016)<br>-Work adjustment was not significantly associated with any of cognitive variables or baseline YMRS, HAM- D or BPRS total score, or other baseline demographic or clinical variables	4 (b,c,d,e)
**18**	Dion et al.<br>1988 (96)	65 patients with BD (manic, mixed or	Longitudinal, (6 month)	**Employment status:**<br>- overall 43% were employed (64% of		2 (d,e)

	USA	atypical)		patients with with 1 admission to hospital and only 33% with multiple admissions)<br>- only 21% of employed worked at expected level of employment<br>- 30% patients was unable to work, even if 80% was with symptomatic recovery		
**19**	Drakopoulos et al. 2020 (66)<br><br>Sweden	120 partially or fully remitted BD-I and BD-II patients<br><br>Employed:<br>Mean age - 35.3<br>MADRS – 3.4, YMRS – 1.7<br><br>Unemployed:<br>Mean age - 40<br>MADRS – 4.0, YMRS – 2.1	Cross-sectional	**Employment status:**<br>-Employed - 72% (n=86)<br>-Unemployed - 28% (n=34)	**Course of the illness, cognitive performance:**<br>Executive functioning was a more powerful predictor of occupational status in BD patients (p=0.001) than IQ and other clinical factors, including illness severity	4 (a,b,c,d)
**20**	Gardner et al. 2006 (35)<br><br>USA	761 employees with BD<br>Mean age: 41.2<br>F/M (%): 54/46<br><br>229,145 employees without BD<br>Mean age: 40.4<br>F/M (%): 44/56	Retrospective	**Work performance:**<br>**Absenteeism:**<br>-Employees with BD had over 2,5 times more health related work absence days annually (18.9 work-days vs 7.4 days annually) than those without diagnosis of BD (p <0 .05)<br><br>**Work costs:**<br>-Costs of employees with BD were 3,17 times higher ($6836 more), than those without diagnosis of BD (p<0.05)		4 (a,b,c,d)
**21**	Gilbert et al. 2010 (109)<br><br>USA	154 BD-I patients<br><br>Mean age: 44.1	Longitudinal (15-43 months)	**Employment status:**<br>-Working at study baseline and termination (Good stable group) – 46.6% (n=49)<br>- Not working at study baseline and termination (Poor stable group) – 30.4% (n=45)	**Cognitive performance, sociodemographic factors:**<br>Self-reported concentration problems *(*p= 0.03) but not baseline physician-rated concentration and memory problems and years of education (p= 0.004) significantly predicted	3 (a,d,e)

				-Not working at study baseline and working at termination (Improving group) – 14.2% (n=21)<br>-Working at study baseline and not working at termination (Worsening group) – 8.8% (n=13)	employment trajectory and may serve as a sensitive predictor of functional outcome in patients diagnosed with BD-I	
**22**	Goldberg et al. 2004 (47)<br><br>USA	BD-I manic (n=34) psychotic (UD (n=17) non psychotic UD (n=72)<br><br>Mean age: 23.2<br>F/M (%): 79/21	Longitudinal, (10 years)	**Work performance:**<br>- Unipolar non psychotic depressed patients had consistently better work functioning than BD at 2-, 7.5 and 10 year follow-up (all p<0.05)<br>-Only 56–64% of the BD patients were functioning effectively in their primary work role for at least half the time over the 10-year period as compared to 80% or more of the nonpsychotic UD patients		4 (b,c,d,e)
**23**	Goldberg et al. 2011 (48)<br><br>USA	95 patients:<br><br>46 -BD-I<br>Mean age (at baseline): 24.3<br>F/M (%): 48/52<br>Symptoms severity (based on Schedule for Aă ective Disorders and Schizophrenia - SADS): Depressive symptoms -49% (n=22), Manic symptoms - 30% (n=13)<br><br>49-non psychotic UD<br>Mean age (at baseline): 23.2<br>F/M (%): 65/35<br>Symptoms severity (based on SADS): Depressive symptoms -40% (n=19)	Longitudinal (15 years)	**Employment status:**<br>BD:<br>Employed - 50% (n=23)<br>UD:<br>Employed - 87% (n=39)<br>**Work performance:**<br>-Work disability was significantly more extensive in BD than UD subjects (p<0.001)<br>**-**BD subjects had lower mean Strauss-Carpenter work functioning ratings (2.3) as compared to UD subjects (3.4), p =0.001	**Symptoms, course of the illness, subsyndromal symptoms, sociodemographic factors:**<br>- Good longitudinal outcome (15years) was significantly predicted by a UD rather than BD diagnosis (p=0.022), and the absence of a depressive syndrome in the past year (p<0.001)<br>-Past- year depressive (p= 0.008),but not past- year manic syndromes (p=0.778) were associated with greater work disability<br>- Subsyndromal depression was significantly associated with poorer work outcome among BD, but not UD subjects<br>-No significant associations were observed with age or the absence of alcohol or drug abuse in the past year (p>0.50)	4 (b,c,d,e)

**24**	Grande et al.<br>2013 (108)<br><br>Spain	327 euthymic patients diagnosed with BD-I or BD-II<br><br>Employed:<br>Mean age: 41<br>F/M (%): 52/48<br>Symptoms severity:<br>MADRS – 4.9<br>YMRS – 2.2<br><br>Unemployed:<br>Mean age: 49<br>F/M (%): 53/47<br>Symptoms severity:<br>MADRS – 7.0<br>YMRS – 3.0	Longitudinal (1 year)	**Employment status:**<br>Employed: 38.7%	**Course of the illness, comorbid disorders, sociodemographic factors:**<br>-Occupational disability in BD patients is associated with axis II comorbidity (p=0.013), more previous manic episodes (p<0.001), not having a stable relationship (p=0.004), older age (p<0.001), and more recurrences at 1-year follow-up (p=0.002)	5 (a,b,c,d,e)
**25**	Hakulinen et al.<br>2019 (49)<br><br>Denmark	Nationwide cohort study including (n = 2,390,127), 1% and 12% with BD and MDD, respectively<br><br>Hospital-based diagnoses of BD and MDD before age 25	Prospective	**Employment status:**<br>- At age 30, 62% of BD and 53% of depression cases were outside the workforce (compared to 19% of the general population)<br>**Work performance:**<br>- Compared to individuals without mood disorders, those with depression and particularly BD had consistently poor socioeconomic outcomes across the entire work-life span<br>**Earnings:**<br>- Individuals with BD or depression earned around 36% and 51%, respectively, of the income earned by individuals without mood disorders	**Course of the illness, sociodemographic factors:**<br>- 52% of BD and 42% of depression cases had no higher education (compared to 27% of the general population)<br>-All associations were smaller for individuals not rehospitalized after age 25	4 (a,c,d,e)
**26**	Haro et al.<br>2011 (113)<br><br>Spain	1656 patients with BD-I<br>Mean age: 44.9<br>F/M (%): 58/42<br>Symptoms severity (at baseline): 4.65<br>(based on Clinical	Longitudinal (2 year)		**Symptoms, course of the illness:**<br>- Impairment of work was consistently associated with lower remission and recovery rates	5 (a,b,c,d,e)

		Global Impression—Bipolar Disorder)			
**27**	Hirschfeld et al.<br>2003 (27)<br><br>USA	600 with BD<br><br>F/M (%): 64/36	Retrospective survey	**Employment status:**<br>-Employed (full- or part-time or student): in 1992: 53%, in 2000: 42%<br>Unemployed: in 1992:46%, in 2000: 58%<br>**Work performance:**<br>- Individuals with BD reported that the illness has a profoundly negative effect on careers and employement	3 (a,b,d)
**28**	Javaras et al.<br>2017 (42)<br><br>USA	Individuals treated in mental health care with BPD (clinically-based individuals with BPD) (n=61)<br>Mean age: 22.5<br>F/M (%): 100/0<br><br>Individuals with BPD community-based (n=164)<br>Mean age: 28.9<br>F/M (%): 88/12<br><br>Individuals without BPD (n=901)<br>Mean age: 36.9<br>F/M (%): 72/28	Cross-sectional survey	**Employment status:**<br>Employed or student:<br>Clinically-based individuals with BPD - 33.8%<br>Community-based individuals with BPD – 77.9%<br>Individuals without BPD – 82.8%<br><br>**Work performance:**<br>- Community-based individuals with BPD had poorer educational/occupational performance as compared to individuals without BPD (77% vs 91%, p=0.03)	4 (a,b,c,d)
**29**	Jovev et al.<br>2006 (41)<br><br>Australia	23 participants with BPD<br>Mean age: 36.2<br>F/M (%): 57/43<br><br>44 with other personality disorders<br>Mean age: 39.3<br>F/M (%): 55/45	Retrospective	**Work performance:**<br>- BPD group reported the poorest levels of functioning, particularly in financial domains and found employment circumstances particularly stressful and difficult to cope with	2 (b,c)

		30 with disorders from axis I (major depression, social phobia, OCD)<br>Mean age: 40.6<br>F/M (%): 50/50				
**30**	Juurlink et al. 2018 (43)<br><br>Netherlands	3672 workers with borderline personality symptoms<br><br>Age: 21-64 years old<br>F/M (%): 43/57	Cross-sectional survey	**Work performance:**<br>-Borderline personality symptoms were associated with low decision latitude, job insecurity and low co-worker support<br>**Absenteeism:**<br>-Borderline personality symptoms were associated with impaired work performance, assessed in total work loss days (the mean of total work loss days in the last 30 days was 2.0)		2 (a,c)
**31**	Jurlink et al. 2019 (19)<br><br>Netherlands	15 with BPD	Retrospective		**Symptoms, course of the illness:**<br>- Barriers to employment were related to characteristics of BPD (such as low self-image, difficulty posing personal boundaries, difficulty regulating emotions, and lack of structure), stigma and support to employment<br>- Enhancing emotion regulation and self-reflection by successful treatment was suggested as a facilitator to enhance employment	1 (b)
**32**	Kaya et al. 2007 (110)<br><br>Turkey	23 BD patients with mania<br>20 with bipolar depression<br>22 healthy controls (total=65)	Cross-sectional		**Subsyndromal symptoms, cognitive performance:**<br>- BD patients recovering from depressive episode may experience more occupational and competency problems due to residual depressive symptoms and impairment of attention and memory	2 (b,c)
**33**	Kessler et al. 2006 (50)	3,378 workers (1.1% with BD-I or II	Retrospective	**Work performance:**<br>**Absenteeism:**	**Symptoms, course of the illness:**<br>-Higher work loss was associated	3 (a,c,d)

	USA	and 6.4% with MDD)<br><br>F/M (%): 46/54		-BD and MDD were associated with significantly elevated annual lost workdays (65.5 and 27.2, respectively) (assessed in the WHO Health and Work Performance Questionnaire - HPQ)<br>**Presenteeism:**<br>-Even more important than absenteeism was presenteeism (35.3 days/year for BD and 18.2 for MDD)<br>**Work costs:**<br>Annual capital loss per ill worker was estimated at $9619 for BPD and $4426 for MDD	with BD than with MDD due to more severe and persistent depressive episodes in those with BD than in those with MDD	
**34**	Landaas et al. 2012 (54)<br><br>Norway	586 clinically diagnosed adult patients with ADHD<br>Age: 34<br>F/M (%): 48/52<br><br>721 controls<br>Age: 29.6<br>F/M (%):59/41	Cross-sectional		**Comorbid disorders:**<br>- Among ADHD patients, cyclothymic temperament was strongly associated with lower educational and occupational achievements and increased psychiatric comorbidity, including BD (10%)<br>- Cyclothymic temperament is highly prevalent in adults with ADHD, and this characterises a subgroup of more psychiatrically impaired individuals, possibly reflecting an underlying affective instability with a pathophysiology closer to the bipolar spectrum disorders	3 (a,b,c)
**35**	Lawrence et al. 2013 (79)<br><br>US	1,051 currently employed with diagnosed MDD<br><br>Mean age: 47<br>F/M (%): 58/42<br>Symptoms severity (based on PHQ-9):<br>no symptoms: 40.2% (n=423)	Cross-sectional survey		**Cognitive performance, symptoms:**<br>-Increased severity of depression symptoms was associated with worsening perceived cognitive functioning (in particular attention/concentration and planning/organisation) ($p < 0.0001$)	3 (a,b,c)

		mild-moderate: 52.9% (n=556)<br>severe: 13.6% (n=143)				
**36**	Lee et al. 2015 (115)<br><br>Australia	Patients with MDD (n=71)<br>Mean age: 19.9<br>F/M (%): 45/55<br>Symptoms severity: depression: 15.1, mania: 9.8 (based on BPRS):<br><br>Patients with BD (n=61)<br>Mean age: 22.8<br>F/M (%): 41/59<br>Symptoms severity: depression:5 mania: 4 (based on BPRS):<br><br>Schizophrenia-spectrum disorders (n=35)<br><br>Healthy controls (n=63)<br>Mean age: 24.1<br>F/M (%): 35/65	Longitudinal (mean 20.6 months)		**Cognitive performance:**<br>- Improved sustained attention was independently associated with	4 (b,c,d,e)
**37**	Lerner et al. 2004 (38)<br><br>USA	Employees with depression (n=85) or dysthymia (n=59)<br>Mean age: 39.8<br>F/M (%): 86/14<br>Symptoms severity: dysthymia – 9.7, MDD – 14.2 (based on PHQ-9):<br><br>173 control group<br>Mean age: 41.4<br>F/M (%): 73/27<br>Symptoms severity 4.2 (based on PHQ-9)	Observational (6 months)	**Employment status at follow-up:**<br>**-** Employment rate: 86% (n=51) – dysthymia, 88% (n=75) – MDD, 98% (n=169) - controls<br>- Unemployment rate:14% (n=8)- dysthymia, 12% (n=10) -MDD, 2% (n=4) - controls<br>**Work performance:**<br>-Patients with depression had significantly more job turnover than with dysthymia and controls (20% vs 13% vs 5%, respectively)<br>**Absenteeism:**<br>-Absenteeism (days missed from work in the past 2 weeks) was higher in		5 (a,b,c,d,e)

		87 group with rheumatoid arthritis<br>Mean age: 47.7<br>F/M (%): 87/13<br>Symptoms severity 2.9 (based on PHQ-9)		dysthymia and MDD group as compared to controls (1.2, 1.6, 0.5, respectively, p<0.001)<br>**Presenteeism:**<br>-Presenteeism (percent productivity lost) was higher in dysthymia and MDD group as compared to controls (5.8, 7.2, 2, respectively, p<0.001)<br>**Earnings:**<br>- Percentage of participants whose incomes increased was significantly greater in control groups than in the depression groups (p=0.027)		
**38**	Martinez-Aran et al. 2007 (104)<br><br>Spain	46 euthymic BD patients with high occupational functioning<br>Mean age: 38.4<br>F/M (%): 63/37<br>Symptoms severity: HDRS: 3.2, YMRS:1<br><br>31 euthymic BD patients with low occupational functioning<br>Mean age: 39.5<br>F/M (%): 58/42<br>Symptoms severity: HDRS: 4.1, YMRS:1.6<br><br>35 controls<br>Mean age: 39.1<br>F/M (%): 63/27<br>Symptoms severity: HDRS:1.8, YMRS:0.8	Cross-sectional	**Employment status:**<br>**-** Unemployed: 90% (n=27) – low occupational functioning, 43% (n=20) – high occupational functioning	**Cognitive performance:**<br>-BD patients showed poorer cognitive performance than healthy controls in particular on verbal memory and executive function measures<br>-Better neuropsychological performance was found in the ‘good occupational adaptation’ group compared with the ‘low occupational adaptation’ group<br>- The best predictor of ‘good occupational functioning’ in BD patients was verbal memory	2 (b,c)
**39**	Martino et al. 2017 (57)	55 with BD-I or BD- II<br><br>Mean age: 43.6	Longitudinal, (mean follow-up 77 months)	**Employment status:**<br>-Employed (full-time): baseline – 39%, at follow up – 44%	**Symptoms, course of the illness:**<br>- Patients with more than 5 previous affective episodes exhibited poorer	4 (b,c,d,e)

	Argentina	Symptoms severity:<br>HDRS:1.65,<br>YMRS:0.56		-Employed (part-time): baseline – 24%, at follow up – 33%<br>-Unemployed: baseline – 37%, at follow up - 22%	outcomes on occupational status than patients with less than 5 episodes, however along a follow-up period measures of functioning tended to remain stable or improved slightly<br>- Functional outcome did not deteriorate over the course of BD	
**40**	McIntyre et al. 2008 (51)<br><br>Canada	20 747 individuals screening positive for lifetime BD-I and MDD (2.4% and 11.2%, respectively)	Retrospective survey	**Employment status:**<br>Employment rate:<br>⌈ BD - 68%,<br>⌈ MDD -69%<br>⌈ no history of mood disorder - 70%<br>**Earnings:**<br>-Individuals with BD or MDD had a significantly lower mean annual income, compared to healthy controls ($p < 0.05$)<br>-Individuals with BD had a significantly lower annual income ($p < 0.05$) and greater odds of mental health disability days in the past two weeks as compared to MDD individuals		3 (a,b,c)
**41**	McMorris et al.<br>2009 (29)<br><br>US	219 BD-I subjects<br>198 control group	Cross- sectional survey	**Earnings:**<br>-BD-I subjects less frequently reported receiving pay for work<br>**Work performance:**<br>-Higher percentage of BD-I subjects reported having been fired or laid off from a job<br>-BD-I subjects report working a reduced schedule for a medical reason<br>-BD-I subjects reported receiving more short-or long-term disability benefits		3 (a,b,c)
**42**	Medard et al. 2010 (105)<br><br>France	31 BD patients<br><br>Mean age: 42.7<br>F/M (%): 62/38	Longitudinal, (3 years)	**Employment status:**<br>-58% were employed in 2003, in 2006 on follow up - 54%	**Comorbid disorders:**<br>-Presence of an associated personality disorder was significantly more frequent in unemployed BD patients, poorer	3 (b,d,e)

					occupational outcome and a lower rate of "return to work"even after adjustment for age and gender (p=0.02)	
**43**	Michalak et al. 2007 (33)<br><br>Canada	Patients with BD (n = 35)<br>Mean age: 43.1<br>F/M (%): 67/33<br>Symptoms:<br>euthymia – 36%<br>depression – 36%<br>mania/hypomania – 24%<br>mixed state – 3%<br><br>and their caregivers (n = 5) and healthcare professionals (n = 12)	Retrospective	**Employment status:**<br>-Employed (full- or part-time or student): 36%<br>Unemployed: 60%<br>**Work performance:**<br>-Five main themes were identified among BD patients: lack of continuity in work history, loss, illness management strategies in the workplace, stigma and disclosure in the workplace, and interpersonal problems at work		2 (b,d)
**44**	Miller et al. 2018 (59)<br><br>Australia	199 subjects with BDP<br>Mean age: 35.2<br>F/M (%): 73/27<br>Participants were highly symptomatic; average number of DSM BPD symptoms met was 8.16	Prospective (12 months)		**Symptoms, course of the illness:**<br>- Those who experienced more severe emptiness, impulsivity and self-harm had worsen outcomes<br>- A relationship between chronic emptiness and impaired vocational outcome at follow-up was found	4 (a,b,c,e)
**45**	Montoya et al. 2010 (68)<br><br>Spain	398 patients with BD<br><br>Mean age: 46.1<br>F/M (%): 56/44<br>Symptoms severity:<br>HDRS:2.5, YMRS:1.4	Prospective (3 years)	**Employment status:**<br>-38.9% of patients with a normal functioning and free of subsyndromal symptoms were on full-time employment<br>- 16-17% patients without normal functioning and/or subsyndromal symptoms were on full-time employment	**Subsyndromal/residual symptoms:**<br>-Syndromal remission was not always accompanied by normal functioning and/or the presence subsyndromal symptoms<br>-Interventions should go beyond syndromal remission and target subsyndromal symptoms and functional recovery	5 (a,b,c,d,e)
**46**	Morriss et al. 2013 (103)	253 patients with BD (238 - BD-I and 15 – BD-II)	Longitudinal, (72 weeks)	**Employment status:**<br>Employed - 60% (n=153)	**Symptoms, course of the illness:**<br>- Strong and stable association between the severity of depressive	5 (a,b,c,d,e)

	UK	Mean age: 41<br>F/M (%): 65/35<br>Symptoms (at baseline):<br>euthymia – 68%<br>depression – 23%<br>mania/hypomania – 6%<br>mixed state – 3%			symptoms and impaired work adjustment correlation coefficients (0.6–0.7)<br>- Severity of mania symptoms was weakly and inconsistently associated with impairment in work adjustment	
**47**	Morselli et al. 2004 (20)<br><br>France, Italy, Holland, Portugal, Russia, Scotland, Spain and Sweden	968 respondents with BD, GAMIAN-Europe/BEAM survey<br><br>Mean age: 44.7<br>F/M (%): 59/41	Retrospectivesurvey,<br>cross- national analysis	**Employment status:**<br>**-Employed** -the percentage varied from 27% in Scotland to 56% in Italy and Portugal<br><br>**-Unemployed** (unemployed or engaged in charity work) - the percentage varied from 6.5 % in Sweden to 34% in France, with globally mean 21.5%<br>- BD leads to a very high rate of unemployment and has a significant negative impact on the perception of the quality of life		3 (a,b,d)
**48**	Mur et al. 2009 (67)<br><br>Spain	44 euthymic BD lithium-treated outpatients<br><br>Employed:<br>Mean age: 39.7<br>F/M (%): 54/46<br>Symptoms severity:<br>HDRS:2.0 , YMRS:2.2<br><br>Unemployed:<br>Mean age: 46.1<br>F/M (%): 45/55<br>Symptoms severity:<br>HDRS:1.6 , YMRS:1.4	Retrospective	**Employment status:**<br>-Employed - 54.5% (n=24)<br>-Unemployed - 45.5 (n=20)	**Symptoms, course of the illness, cognitive performance, subsyndromal or residual symptoms:**<br>- Remission in BD is not synonymous with recovering in psychosocial and occupational functioning<br>- Cognitive deficits, clinical course and persistent subsyndromal symptoms may may particularly affect occupational functioning	2 (c,d)
**49**	O'Donnell et al.<br>2017 (34)	129 participants with BD	Cross-sectional	**Employment status:**<br>-Employed – 53% (n=68)<br>-Unemployed – 47% (n=61)	**Symptoms, course of the illness:**<br>-Higher degrees of depression and conflict at work are associated with	4 (a,b,c,d)

	USA	Employed:<br>Mean age: 51.1<br>F/M (%): 80/20<br>Symptoms severity:10.3 (based on BDI)<br><br>Unemployed:<br>Mean age: 46.5<br>F/M (%): 77/23<br>Symptoms severity:19.4 (based on BDI)		**Work performance:**<br>-Greater stigma and exclusion at work were associated with unemployment among BD individuals (both p<0.05)	work impairments for employed individuals (p<0.05)	
**50**	O'Shea et al. 2010 (60)<br><br>UK	29 euthymic BD patients<br>Mean age: 51.7<br>F/M (%): 51/49<br>Symptoms severity: HDRS:3.3 , YMRS:1.6<br><br>29 matched controls<br>Mean age: 54.2<br>F/M (%): 51/49	Cross-sectional	**Employment status:**<br>- Unemployed (among BD patients) – 14% (n=14)<br>**Work performance:**<br>- Patients with BD showed impairment in occupational functioning	**Symptoms, course of the illness, cognitive performance:**<br>-Unemployment was associated with impairment in attention<br>-Memory impairment correlated with number of previous manic episodes	2 (b,c)
**51**	Reed et al. 2010 (112)<br>Italy, Spain	n = 2289 BD patients with a manic/mixed episode	Longitudinal (2 years)		**Symptoms, course of the illness:**<br>-Work impairment in BD is associated with hospital admission, rapid cycling	4 (a,b,d,e)
**52**	Rosa at al. 2009 (107)<br><br>Spain	71 euthymic BD patients<br>Mean age: 47.7<br>F/M (%): 49/51<br>Symptoms severity: HDRS:2.12, YMRS:1.04<br><br>61 healthy controls	Cross-sectional		**Symptoms, course of the illness, sociodemographical factors:**<br>- Depressive symptoms, older age, number of previous mixed episodes, and number of previous hospitalisation were associated with poor occupational functioning	3 (b,c,d)
**53**	Ruggero et al. 2007 (46)<br><br>USA	Total 1385 patients with depression:<br><br>45 with BD-I<br>Mean age: 34.7	Cross-sectional retrospective	**Work performance:**<br>- BD II disorder was associated with serious work impairment and a high number of serious suicide attempts - the level of impairment was more similar		3 (a,c,d)

		F/M (%): 67/33<br>Symptoms severity: 2.93<br>(based on CGI-S)<br><br>89 with BD-II<br>Mean age: 35.9<br>F/M (%): 61/39<br>Symptoms severity: 2.83<br>(based on CGI-S)<br><br>1251 with MDD<br>Mean age: 38.8<br>F/M (%): 66/34<br>Symptoms severity: 2.88<br>(based on CGI-S)		than it is different from that in BD-I<br>-Clinicians would be mistaken to presume that the "softer" bipolar spectrum, specifically BD-II is less impairing than BD-I<br>**Absenteeism:**<br>-BD-I and BD-II disorder were associated with greater absenteeism from work (more than 1 year) compared to MDD (58% vs 33% vs 16%, respectively, p<0.05)		
**54**	Ryan et al. 2013 (111)<br><br>USA	156 euthymic BD patients<br><br>Employed:<br>Mean age: 36.1<br>F/M (%): 42/58<br>Symptoms severity: 2.88<br>HDRS:4.1, YMRS:1.5<br><br>Unemployed:<br>Mean age: 42.2<br>F/M (%): 39/61<br>Symptoms severity:<br>HDRS:4.8, YMRS:2.0<br><br>143 controls	Observational, cross-sectional		**Cognitive performance, symptoms, course of the illness:**<br>-Emotion processing and executive tasks were predictive of BD unemployment, after accounting for number of mood episodes<br>-BD participants in the not working group had greater number of mood episodes<br>- There were no other significant differences between the working and not working BD participants on other clinical variables, including current mood symptoms (p<0.05)	4 (a,b,c,d)
**55**	Samalin et al. 2016 (100)<br><br>France	468 euthymic patients with BD<br><br>Mean age: 47.7	Observational, cross-sectional, multicenter	**Employment status:**<br>-Employed - 48.5% (n=227)	**Subsyndromal /residual symptoms:**<br>- Negative effects of residual manic symptoms and occupational stigma	3 (a,b,d)

		F/M (%): 59/41			on autonomy and financial issues<br>- Negative effects of residual depressive symptoms and emotional inhibition on occupational functioning	
**56**	Sansone et al. 2006 (53)<br><br>USA	94 internal medicine outpatients (28 meeting criteria for borderline personality symptomatology- BPS)<br><br>Mean age: 41.8<br>F/M (%): 61/38	Retrospective, survey		**Sociodemographical factors:**<br>-There may be a relationship between borderline personality symptomatology and employment disability only among women (r = 0.33, r = 0.36, p = 0.05)	1 (d)
**57**	Sansone and Wiederman 2013 (44)<br><br>USA	1493 individuals with borderline personality symptomatology (BPS)<br><br>Mean age: 50.7<br>F/M (%): 67/33	Cross-Sectional, survey	**Work performance:**<br>- 7.6% of participants endorsed losing a job on purpose and those participants scored statistically significantly higher on measures of borderline personality symptomatology and this was independent of gender or age<br>- One factor in poor employment viability among individuals with BPS appears to be the behaviour of losing a job on purpose		3 (a,b,d)
**58**	Schoeyen et al. 2013 (64)<br><br>Norway	226 BD patients<br><br>Mean age: 33.9<br>F/M (%): 62/38<br>Symptoms severity: YMRS:3.4	Longitudinal (7 years)		**Symptoms, course of the illness, cognitive performance:**<br>-Occupational outcome was unrelated to premorbid and current IQ, as well as decline in IQ<br>-Persistence of severe clinical symptoms, rather than global cognitive functioning, determines occupational outcome in BD	5 (a,b,c,d,e)
**59**	Shippee et al. 2011 (30)<br><br>USA	Total=59 941 individuals<br>572 patients with BD<br>5 464 with depression<br>53 905 controls	Cross-sectional, survey	**Employment status:**<br>-Employed: BD – 46%, MDD – 63%, Controls – 81%<br>-Unemployed: BD – 57%, MDD – 37%, Controls – 19%<br>**Work performance:**		4 (a,b,c,d)

				- BD or depression patients were more likely to be unemployed, miss work, and have cognitive, limitations than controls<br>**Absenteeism:**<br>Average missed days of work: BD – 8.36, MDD – 7.45, Controls – 3,45<br>**Work costs:**<br>- Individuals with depression had higher odds of work-related costs than those with no mood disorder, but their odds ratios were smaller than for BD		
**60**	Simon et al. 2008 (97)<br><br>USA	441 outpatients with BD<br><br>Employed:<br>Mean age: 44<br>F/M (%): 68/32<br>Symptoms:<br>depression – 74%<br>hypomania/mania – 56%<br><br>Unemployed:<br>Mean age: 47<br>F/M (%): 70/30<br>Symptoms:<br>depression – 85%<br>hypomania/mania – 64%	Longitudinal (24 months)	**Employment status:**<br>-Employed – 62.1 % (n=274)<br>-Unemployed – 31.3% (n=138)<br>**Work performance:**<br>**Absenteeism:**<br>- Depression was associated with 4.06 additional days of work missed per month compared to those without significant depressive symptoms	**Symptoms, course of the illness:**<br>- Manic or hypomanic symptoms were not significantly associated with probability of employment<br>- Depression was strongly and consistently associated with decreased probability of employment (15% less) than lack of significant depressive symptoms	5 (a,b,c,d,e)
**61**	Sio et al.<br><br>2011 (58)<br>Australia	60 participants with BPD<br>Mean age: 19<br>F/M (%): 80/20<br>Symptoms severity: (based on Structured Clinical Interview for DSM-IV Axis II Personality Disorders (SCID-II) BPD	Observational (12 months)	**Employment status:**<br>-Employed (or student) – 66.7% (n=40)	**Symptoms, course of the illness:**<br>-Impulsivity was associated with poor vocational outcome after 12 months	4 (b,c,d,e)

		module): mean number of BPD criteria – 5.7				
**62**	Sole et al. 2018 (61)<br><br>Spain	143 euthymic BD in 3 groups: good functioning, moderate functioning and with difficulties in all areas of functioning<br>Mean age: 41.2<br>F/M (%): 58/42<br>Symptoms severity: HDRS: 3.2. YMRS:1.3<br><br>60 healthy controls	Retrospective cross-sectional		**Subsyndromal or residual symptoms, cognitive performance:**<br>- Both functionally impaired groups were characterised by higher sub-threshold symptoms (depressive and manic) and higher unemployment rates<br>- The most functionally impaired group also showed lower scores on some measures of processing speed	4 (a,b,c,d)
**63**	Soloff and Chiapetta 2020 (106)<br><br>USA	150 participants with BPD<br>Mean age (at baseline): 38.1<br>F/M (%): 83/17	Longitudinal, (mean 9.94 years)	**Employment status:**<br>-At follow-up 50.0% had full-time employment or attended school	**Symptoms, course of the illness, comorbid disorders:**<br>- Remission from BPD was neither necessary or sufficient for full-time employment<br>- Comorbidity with anxiety, major depression or substance use disorder predicted poor outcome	4 (a,b,d,e)
**64**	Spalt 1977 (116)	41 – UD<br>19-BD	Longitudinal	**Work performance:**<br>-Bipolar group was more permanently unemployed than depression group		2 (c,e)
**65**	Stang et al. 2007 (26)<br><br>USA	59 patients with BD<br><br>(22% - mixed episode 5% manic-predominant, 12% hypomanic-predominant<br>46%- depressive-predominant episode)	Survey	**Employment status:**<br>-Employed (full- and part-time or student) - 55.9% (n=33)<br>-Unemployed – 40.7%% (n=32)<br>**Work performance:**<br>- 41% reported fearing the loss of their current job due to their emotional state; and 20% reported being fired/laid off during the past 5 years due to their emotional state<br>**Absenteeism:**<br>- BD patients reported problems with employment -50% reported missing at least 1 week of work during the past		3 (b,c,d)

				month		
**66**	Strassnig et al. 2017 (101)<br><br>USA	128 participants with: schizophrenia (n=80) and BD (n=48)<br>Mean age: 47.8<br>F/M (%): 46/54	Prospective (20 years)	**Employment status at follow-up:**<br>-Employment rate among BD individuals – 53.4%	**Symptoms, course of the illness, cognitive performance:**<br>-Employment status was independent from other, previously established predictors of everyday outcomes, including cognition and symptoms	3 (b,d,e)
**67**	Strassnig et al. 2018 (102)<br><br>USA	233 participants with: schizophrenia (n=146) and<br>BD (n=87)<br>Mean age: 47.6<br>F/M (%): 50.5/49.5<br>Severity of symptoms: 4.7 (based on HDRS)	Prospective (10 years)	**Employment status:**<br>-Employment rate among BD individuals – 52.8%		4 (b,c,d,e)
**68**	Tei-Tominaga et al.<br>2012 (55)<br><br>Japan	728 Japanese	Cross-sectional survey		**Comorbid disorders:**<br>-The affective temperaments (cyclothymic and anxious) were high-risk factors for depressive symptoms<br>- Depressive symptoms among Japanese between 20-40 years old (a group in which immature-type depression, classified in BS, is common) may be indicative of immature-type depression, in which cyclothymia is the premorbid personality	2 (a,b)
**69**	Uribe et al. 2017 (39)<br><br>Colombia	107 patients with MDD or double depression (MDD plus dysthymia)<br><br>Mean age: 42<br>F/M (%): 57/43	Retrospective	**Employment status:**<br>-Employed (full- or part-time) – 61%<br>**Work performance:**<br>**Absenteeism:**<br>- Absenteeism was reported by 70% of patients<br>-Number of hours/month lost due to absenteeism was 43<br>**Presenteeism:**		3 (a,b,d)

				- Presenteeism was reported by 99%<br>-Number of hours/month lost due to presenteeism was 51<br>**Work costs:**<br>- The 2015 monetary value of productivity losses amounted to US $840 million		
**70**	Waghorn et al. 2007 (24)<br><br>Australia	156 patients with BD<br>Mean age: 29.5<br>F/M (%): 53/47<br><br>385 with schizophrenia	Cross-sectional survey	**Employment status:**<br>-Employment rate among BD individuals – 26.9% (n=42)<br>-Unemployed – 73.1% (n=114)	**Course of the illness, comorbid disorders, sociodemographic factors:**<br>-Correlates of employment functioning unique for BD patients were: age, course of disorder, lifetime substance dependence and repeated use of illicit drugs	1 (a)
**71**	Wilkins 2004 (72)<br><br>Canada	938 patients with BD-I	Retrospective, survey	**Employment status:**<br>-Employed – 68, 8% (as compared to general population – 77.6%, it is significantly different)	**Symptoms, course of the illness:**<br>- BD -I individuals with major depression were less likely to be employed<br>- Hospitalisation within 12 months reduces odds of being employed	3 (a,c,d)
**72**	Woodhead et al. 2020 (114)<br><br>USA	382 individuals with depression<br><br>F/M (%):56/44	Prospective (23 years)		**Symptoms, course of the illness:**<br>-The high-severity depression course group had the poorest work functioning outcome, followed by the moderate-severity and then the low-severity groups	5 (a,b,c,d,e)
**73**	Zimmerman et al. 2010 (36)<br><br>USA	206 patients with BD-I or BD-II<br><br>Mean age: 35.8<br>F/M (%): 59/41<br>Symptoms:<br>remissions – 77.3%<br>partial remissions – 24.4%<br>depression/mania/mixed state – 68.4%	Retrospective	**Employment status:**<br>-Prolonged unemployment (missing up to 2 years or more of work) - 34.4% (n=71)<br>**Work performance:**<br>**Absenteeism:**<br>- Less than 20% of the patients reported not missing any time from work due to psychiatric reasons, and more than 1/3 missed up to two years or more from work	**Symptoms, course of the illness, comorbid disorders, sociodemographic factors:**<br>- Prolonged unemployment was associated with increased rates of current panic disorder, lifetime history of alcohol abuse or dependence, older age and experienced more episodes of depression	4 (a,b,c,d)

**74**	Zimmerman et al.<br>2012 (73)<br><br>USA	1333 patients with:<br><br>181 MDD with BPD<br>Mean age: 40.1<br>F/M (%): 56/44<br><br>1068 MDD without BPD<br>Mean age: 33.3<br>F/M (%): 71/29<br><br>84 BD<br>Mean age: 36.2<br>F/M (%):56/44	Cross-sectional	**Employment status:**<br>-MDD with BPD:<br>Persistent unemployment (missing up to two years or more) -24.9% (n=45)<br>Chronically unemployed (throughout the five years) – 7.2% (n=13)<br>-MDD without BPD:<br>Persistent unemployment – 10.6% (n=113)<br>Chronically unemployed – 3.4% (n=36)<br>-BD:<br>Persistent unemployment – 25% (n=21)<br>Chronically unemployed – 8.3% (n=7)	**Comorbid disorders:**<br>- MDD with BPD were more likely to be persistently unemployed than without BPD<br>- Both BD and BPD were associated with impaired occupational functioning	5 (a,b,c,d,e)
